# Percutaneous nephrostomy by direct puncture technique: An observational study

**DOI:** 10.4103/0971-4065.65301

**Published:** 2010-04

**Authors:** R. Karim, S. Sengupta, S. Samanta, R. K. Aich, U. Das, P. Deb

**Affiliations:** Department of Radiodiagnosis, Institute of Postgraduate Medical Education & Research (IPGMER), Kolkata, West Bengal, India; 1 Department of Radiotherapy, NRS Medical College, Kolkata, West Bengal, India

**Keywords:** Percutaneous nephrostomy, pigtail catheter, urinary tract obstruction

## Abstract

Percutaneous nephrostomy is the procedure of establishing a temporary drainage tract of the renal pelvi-calyceal system through the skin. This study aims to find out whether low cost trocar catheter can be a suitable substitute for the relatively high cost fluoroscopy/ultrasonography guided tract dilatation and tube insertion procedure. Percutaneous nephrostomy by the trocar catheter was performed in 126 patients. Under local anesthesia, a stab wound deep enough to traverse the muscle layer was made through which the trocar - catheter drainage set was inserted under ultrasonography guidance. About 179 procedures were performed in 126 patients. Primary technical success rate was 94%, major complication rate 1.6%, minor complication rate 11% and catheter related complications like catheter blockage or dislodgement were 13%. There was no procedure related mortality in our series. The ultrasonography-guided trocar, catheter nephrostomy, is a quick, safe and low cost procedure in selected cases of upper urinary tract obstruction. The primary technical success and complication rates are comparable to any other reported procedure and its low cost is particularly suitable for developing countries like India.

## Introduction

Percutaneous nephrostomy is a procedure of establishing a drainage tract into the upper urinary system by puncturing the kidney directly through the skin. The purpose of such drainage is to decompress the upper urinary passage caused as a result of supra or intra vesical obstruction resulting in increased pressure within the collecting system and ultimately renal parenchymal damage. It can be used as a conduit for diagnostic and therapeutic procedures to provide urinary diversion and improve renal functions. The standard ‘Seldinger’ technique uses a needle puncture, guide wire insertion and serial dilatation. In our institute we have resorted to low cost trocar catheter drainage set for the procedure. It is simple, less time consuming, less traumatic and has excellent outcome.

## Materials and Methods

From September 2006 to February 2009, 126 patients with varying degrees of supra or intra vesical obstruction were enrolled in the present study. After obtaining a written request from the concerned physician for percutaneous nephrostomy, stating the reason for such a procedure, the patient was admitted in the hospital. The patients‘ disease profile causing the obstruction has been shown in Table 1. Pre-procedure investigations advised were: routine blood count, prothrombin time, bleeding and clotting time, serum creatinine and electrolytes estimation, urine culture and plain X-ray KUB. As the procedure is done under local anesthesia, all the patients were briefed in detail preoperatively and their co-operation, e.g. suspending the breath at the moment of puncture was emphasized and written informed consent was taken from every patient, counter signed by his next kin.

**Table 1:** Disease profile requiring percutaneous nephrostomy (n=126)

Disease profile	No.
Carcinoma cervix	36
Carcinoma urinary bladder	29
Pyonephrosis	11
Accidental injury during operation	01
BOTO with CRF	08
PUJ obstruction of right kidney	07
PUJ obstruction of left kidney	08
Suspected stricture following stone removal	05
Huge ovarian mass with HDN	05
Unknown etiology	10
Large calculus in mid-ureter	06

A pre-procedural sonography was done to decide the nature and site of obstruction. The minimum measurement of the dilated pelvis was taken to be 20 mm. Pigtail catheter of 6F to 8.5F were chosen depending on the age of the patient, clinical and imaging findings. The patient was advised not to take oral feed for the last six hours. Sedatives and long acting analgesics were given the previous night. Below 12^th^ rib approach in prone position was preferred during the procedure. A supporting pillow was placed under the abdomen on the side of the procedure to correct lumber lordosis and to support the kidney. Local anesthetic (2% Xylocaine) was injected to anesthetize the puncture site after proper dressing and draping of the area. The anesthetic was given past the muscular layer up to the renal capsule. While it was given, future pathway of the catheter is also decided. After local anesthesia, Betadine jelly was applied on the transducer, localization of the puncture site again confirmed and distance between the puncture site and the target calyx measured. A puncture wound was made by a sharp blade (size 11). The stab wound was deep enough to traverse the muscle layer, through which the trocar catheter drainage set was is inserted under ultrasound guidance in a postero-lateral approach. The course of the drainage set and the ultra sound beam was kept parallel for better visualization. No guide-wire or dilatation was undertaken during the procedure. A small portable ultrasound machine with a sector probe of 3MHz was used for tube insertion. The operator monitored the pathway of the tube in real time with his right hand while his left hand inserts and mobilizes the drainage set. Care was taken to see the dilated calyx and the drainage tube in-situ lie in a “single slice”. Most of the time the lower polar calyx was attempted but a few cases (13 out of 179) were done through the upper or middle calyx. Once the drainage set was *in situ*, the trocar with the two inner metallic tubes was taken out leaving the pig-tail catheter *in situ*. Urine was collected for culture and sensitivity test, the wound was sutured, tube fixed and drainage cannula was connected with an urobag through the agency of a suitable adapter. Broad spectrum antibiotic was routinely given pre and post operatively and patients were followed up by urinalysis and clinical examination for the next 48 hours. Serum creatinine estimation was done after 48 hours and again after 14 days.

## Results and Analysis

Of the 126 patients in whom percutaneous nephrostomy was attempted, 72 (57%) were male and 54 (43%) were female. The median age of the male patients was 47 years and in female it was 41 years. The obstruction was due to advanced malignancies in 65 (52%) patients and due to benign conditions in 51 (40%). In 10 (08%) patients, etiology was unknown at the time of nephrostomy. 62 of our patients had bilateral hydronephrosis and in 64 patients it was unilateral. We had two patients with hydronephrosis of the only functioning kidney; the other kidney was removed previously. One of our patients is a postoperative case with both the ureters accidentally ligated during a difficult radical hysterectomy. The patient developed severe septicemia and pyonephrosis. Excellent results were obtained after the drainage procedure and subsequently the patient underwent re-implantation of ureters. We had 11 other patients suffering from pyonephrosis. Eight patients were referred to the urosurgeon for open nephrostomy as we failed to negotiate the trocar catheter in spite of repeated efforts to do so. In five patients it was found out to be due to malrotated kidney(s) in which direction of the drainage set could not be monitored properly. In the remaining three patients no definite reason of failure could be ascertained. Table 2 shows the complication following the procedure. Almost all (108 out of 126) patients complained of local pain of varying degrees, which persisted for two to 48 hours. One patient complained of pain in the right shoulder during the procedure, which typically ended minutes after completion of the procedure. 12 patients complained of macroscopic hematuria for 24-36 hours of which two required bold transfusion but no operative intervention. Nine patients required more effort and developed mild perinephric collections, which resolved spontaneously in next three days. In 24 patients reinsertions were required, as the drainage tips were blocked in 16 patients and putrid smelling sero-sanguineous fluid was aspirated with needles from the calyces. In the rest eight patient dislodgement of the catheter occurred. It was observed that the most common problem during insertion of the drainage cannula is telescoping effect of the sheath, which had to overcome by increasing the dimension of the stab-wound up to the muscle layer. Mean serum creatinine levels were 4.16 mg/dl, 3.49 mg/dl and 2.85 mg/dl before, 48 hours after and 14 days after the procedure. However, changes in serum creatinine levels were more marked in benign than malignant conditions. The average time taken to complete the procedure was 39 minutes, ranging from 25 to 55 minutes.

**Table 2:** Intra and immediate post procedural complications (*n*=126)

Complication	No. (%)
Only pain in loin	108 (86)
Pain in shoulder tip	01 (0.8)
Macroscopic hematuria	12 (9.6)
Perinephric collection	03 (2.4)
Blocked tube/Dislodgement	24 (19)
Fever	13 (10.3)
Insertion failure	08 (6)

** Some patients had more than one complication

## Discussion

Obstructive uropathy results in pain, infection, sepsis and ultimately loss of renal function. It is a potentially life threatening condition and sometimes it is desirable to provide immediate temporary relief of the obstruction, until definitive treatment can be undertaken. Cystoscopy with retrograde catheterization and operative nephrostomy, are two valid options with their own disadvantages. Sometimes it may be impossible to pass the ureteric catheter above the obstructing lesion and even when passed, it is uncomfortable and often becomes dislodged into the urinary bladder. Operative nephrostomy is a major surgical procedure requiring general anesthesia for, what may be a transitory obstruction and already impaired renal function may make it hazardous. On the other hand, percutaneous nephrostomy is a very simple procedure of temporary drainage of an obstructed kidney by establishing a drainage tract into the pelvi-calyceal system of the affected kidney directly through the skin.

Percutaneous nephrostomy was first described by Goodwin *et al*.[1] half a century ago and recently reviewed by Dyer *et al*.[2] Classically it involves fluoroscopy guided puncture followed by tract dilatation and insertion of the tube with or without use of guide wire and a success rate of around 95% has been reported.[3] Other workers[4] have similarly emphasized the role of tract dilatation and subsequent drainage tube placement.

The first ultrasonography-guided percutaneous nephrostomy was performed by Pederson and achieved a success rate of about 70%.[5] Since then, a large number of studies of ultrasound guided percutaneous nephrostomies have been carried out, particularly in the last two decades and a success rate up to 92% have been reported,[6] which is comparable to the fluoroscopy-guided methods without any appreciable radiation hazard. This has been achieved because of the advent of the high resolution ultrasound machines enabling accurate viewing of the pelvi-caliceal system. In our study, primary technical success rate was 94% which is very much comparable with the results of Nielson[6] and Kehinde.[3] The high success rate was probably due to proper patient selection as some degree of dilatation of the pelvi-caleceal system was incorporated in the patient selection criteria.

Ogg[7] has summarized the purpose of percutaneous nephrostomy as (1) short term relief of patients in anuria (2) short term effect in patients awaiting pyeloplasty, particularly in patients with high risk of pyonephrosis and (3) assessment of potential functional recovery of an obstructed kidney. Recent guidelines issued by the American College of Radiologists[8] have covered all the relevant aspects, including current indications.

The present procedure is actually a departure from the standard protocol, started in our department in a desperate attempt to salvage a kidney of a chronically obstructed case of bilateral hydronephrosis, who could not purchase the standard set costing approximately INR 5000 / at that time. Being encouraged with its success, the facility was extended to other poor patients. Early decompression and consequent functional recovery improves the patients’ condition to a great extent with considerable impact on the final outcome. Patient selection, operators’ skill, relative contra-indications and preliminary base line investigations are the keys to success. Most patients tolerate the procedure well under local anesthesia. Less than 10 ml of 2% lignocaine was used in our series. The use of adrenaline with 1% lignocaine is widely practiced and are said to increase the safety by reducing some of the tract bleeding. We have never attempted to use adrenaline in the current series as tract dilatation was not a part of our procedure. Percutaneous nephrostomy under ultrasound- guidance is very reliable and precise, only limiting factors being the skill of radiologist and some degree of dilatation of the pelvi-calyceal system. After we had become familiar with the technique, the total procedure could be accomplished in less than 30 minutes. In a dilated system the posterior lower pole calyx is easily identifiable from a below 12^th^ rib approach. Higher calyces are more difficult to be discerned individually. Locating and puncturing a dilated calyx is straightforward but correct placement of catheter could be a bit difficult.

Obstruction of the catheter may occur, and a second catheter may successfully be inserted when this happened. A possible cause of such obstruction is kinking due to excessive coiling in the renal pelvis though Ogg[7] had opined that excessive coiling does not cause kinking particularly when ‘woven’ catheters are used.

Cases with pretreated advanced malignant disease involving both ureters raise ethical problems, as the benefits resulting from relief of the obstruction is likely to be temporary and no definite management is available for the etiological factor. Sood *et al*.[9] found no significant recovery of renal functions after percutaneous nephrostomy in their malignant cohort of patients. So did Somarsinghe *et al*.[10] in patients with chronic obstruction and terminal malignancy. In our patients also, the decrease of the serum creatinine levels were not as marked as in benign conditions but there was some definite improvement of the quality of life even when the obstruction was due to advanced malignancy. However, the ultimate decision lies with the patient himself. But in other cases of anuria due to obstruction, it is advantageous as it improves the general condition of the patient and helps to reduce the risk of infection before definitive surgery is contemplated. In unilateral obstructive disease there is an interesting further possibility, since a period of satisfactory drainage makes it possible to assess the likely return of function of the kidney with adequate relief of obstruction.

When an asymptomatic patient presents with a nonfunctioning and obstructed kidney, a temporary nephrostomy preserves any recoverable renal tissue and allows accurate measurement of the differential glomerular filtration rate. Many a times a seemingly alarming radiological appearance where little functional recovery is expected, dramatic improvement in renal function has been noticed. So was the observation of Dyer *et al*.[2] Unnecessary and occasionally tragic nephrectomies could thus be avoided. The final outcome still requires further assessment. Lastly, majority of these patients who require a percutaneous nephrostomy presented with some form of obstruction in their urinary system. Even after the appropriate treatment residual obstruction might still be present: e.g. a stricture at the site of impaction after the stone was removed. In such situation, a temporary drainage procedure gives adequate restoration of renal function for undertaking future antegrade dilation and relief of the stricture. In one such case the patient required multiple efforts of dilatation, during which she came for regular follow-up up to three months with the drainage tube *in situ*. In this series, there was no mortality and significant complication like macroscopic hematuria occurred in only 1.6% of our patients. Though procedure related mortality of percutaneous nephrostomy is very low, but a figure up to 0.3% and significant bleeding requiring transfusion or surgical intervention in 1 - 3% has been reported.[11] In a large series of 1207 percutaneous nephrostomies, Stables[12] reported a 4% significant complication rate.

The very low rate of significant hemorrhage in our patients is probably related to two factors: (i) puncture site selection - we have chosen a puncture site two cm below the 12^th^ rib near posterior axillary line and an oblique postero-lateral approach along the Brodel line into the end of a posterior calyx (ii) absence of tract dilatation. The two most important criteria that we learnt from our present exercise are: proper selection of patients and perfect visualization of the dilated calyx with *in situ* catheter in a single slice are keys to success. Another important learning is the ability of the patient to co-operate for successful outcome. In the sole pediatric case (aged one year with bilateral hydronephrosis, a shot of ketamine was used), the drainage set was inserted after two postponements. A number of authors have reported that post procedure fever for the next 48-hours or so is not uncommon. We could not corroborate this fact, primarily because we have observed fever only in those cases that were suffering from pyonephrosis or where tubal blockage led to some form of infection and we routinely used peri - procedural antibiotics. With experience, adequate planning and careful patient selection, percutaneous nephrostomy is a very safe procedure in skilled hands. With a reported mortality of up to 0.3%, it renders open nephrostomy obsolete as an isolated procedure. Further, the pathway it allows to the inside of the kidney opens up the whole new field of endourology. There is paucity of reports where percutaneous nephrostomy has been done with pigtail catheter without help of the guide-wires and dilators. In our series direct visualization by real time sonography ensured correct placement of the catheter.

Percutaneous nephrostomy can be performed on an outpatient basis in selected patients.[9,13] Patients who live alone or in whom the risk of complication is high, such as those with ‘stag horn’ calculi, uncorrected hypertension or a coagulopathy, are best treated in an in-patient setting for proper monitoring for at least 48 hours.

## Conclusion

Ultrasound guided Percutaneous nephrostomy by direct puncture set is a reliable, easy, cost effective technique for making a temporary drainage pathway in case of supra or intra vesical urinary obstruction. For a skilled hand, complication rates are negligible. This procedure is particularly suitable for developing countries like India, where majority of patients attending these hospitals are of low income group and infrastructural facilities are too inadequate to cope up with the vast patient load. Open nephrostomy should be reserved only for those cases in which it is definitely indicated or; percutaneous nephrostomy could not be carried out due to some other reasons.
